# Digital Self-monitoring of Multiple Sclerosis: Interview Study With Dutch Health Care Providers on the Expected New Configuration of Roles and Responsibilities

**DOI:** 10.2196/30224

**Published:** 2022-04-27

**Authors:** Karine Wendrich, Lotte Krabbenborg

**Affiliations:** 1 Institute for Science in Society Radboud University Nijmegen Nijmegen Netherlands

**Keywords:** digital self-monitoring, smartphone apps, multiple sclerosis, technology assessment, health care providers, user participation, mobile phone

## Abstract

**Background:**

Digital self-monitoring allows patients to produce and share personal health data collected at home. This creates a novel situation in which health care providers and patients must engage in a reconfiguration of roles and responsibilities. Although existing research pays considerable attention to the perceptions of patients regarding digital self-monitoring, less attention has been paid to the needs, wishes, and concerns of health care providers. As several companies and public institutions are developing and testing digital self-monitoring at the time of writing, it is timely and relevant to explore how health care providers envision using these technologies in their daily work practices. Our findings can be considered in decision-making processes concerning the further development and implementation of digital self-monitoring.

**Objective:**

This study aims to explore how health care providers envisage using smartphone apps for digital self-monitoring of multiple sclerosis (MS) in their daily work practices, with a particular focus on physician-patient communication and on how health care providers respond to self-monitoring data and delegate tasks and responsibilities to patients.

**Methods:**

We conducted semistructured in-depth interviews with 14 MS health care providers: 4 neurologists, 7 MS specialist nurses, and 3 rehabilitation professionals. They are affiliated with 3 different hospitals in the Netherlands that will participate in a pilot study to assess the efficiency and effectiveness of a specific smartphone app for self-monitoring.

**Results:**

The interviewed health care providers seemed willing to use these smartphone apps and valued the quantitative data they produce that can complement the narratives that patients provide during medical appointments. The health care providers primarily want to use digital self-monitoring via prescription, meaning that they want a standardized smartphone app and want to act as its gatekeepers. Furthermore, they envisioned delegating particular tasks and responsibilities to patients via digital self-monitoring, such as sharing data with the health care providers or acting on the data, if necessary. The health care providers expected patients to become more proactive in the management of their disease. However, they also acknowledged that not all patients are willing or able to use digital self-monitoring apps and were concerned about the potential psychological and emotional burden on patients caused by this technology.

**Conclusions:**

Our findings show that health care providers envisage a particular type of patient empowerment and personalized health care in which tensions arise between health care providers acting as gatekeepers and patient autonomy, between patient empowerment and patient disempowerment, and between the weight given to quantitative objective data and that given to patients’ subjective experiences. In future research, it would be very interesting to investigate the actual experiences of health care providers with regard to digital self-monitoring to ascertain how the tensions mentioned in this paper play out in practice.

## Introduction

### Background

Increasing attention is being paid to digital self-monitoring by patients, that is, patients using digital devices to collect and record personal health data on bodily functions and everyday activities, such as various physical activities, mental status, and sleep patterns [1,2]. Smartphone apps, which are software programs designed for mobile devices, can be used for digital self-monitoring [3,4]. These apps make it relatively easy to produce and share personal health data and can therefore facilitate the maintenance of health and the self-management of chronic conditions [4]. Digital self-monitoring has implications for current care practices, as the setting for care is shifting from the hospital to the home, leading to new roles and responsibilities for health care providers as well as for patients [5,6]. There is considerable interest in using digital self-monitoring technologies to facilitate monitoring and self-management of multiple sclerosis (MS), a chronic neurological disease [7,8]. MS is characterized by a high variability of unpredictable symptoms, and the disease course is also unpredictable. Common symptoms are fatigue, mobility issues, and cognitive problems [9,10]. Digital self-monitoring is thought to support people with MS to self-manage their health in their home setting, for instance, by monitoring symptoms and adapting lifestyle behaviors [7,11-13].

Although existing research pays considerable attention to the motivations and perceptions of patients regarding digital self-monitoring, less attention has been paid to the needs, wishes, and concerns of health care providers [13,14]. Our research fills this gap and focuses particularly on how health care providers envision their new roles and responsibilities. Several scholars in the field of medical sociology (eg, the studies by Krabbenborg [15], Oudshoorn [16], Burri [17], and Pols [18]) have already shown how the introduction of new medical technologies in existing care infrastructures, be they eHealth technologies or otherwise, destabilizes the roles and responsibilities of health care providers. Burri [17], for example, showed how new digital imaging technologies challenged the identity and expertise of radiologists, as they had to acquire new knowledge and skills to interpret the images and use the machines [17]. Because their expertise was questioned by the introduction of these new technologies, radiologists were forced to re-establish their position as visual experts within the medical field. Pols [18] discussed how telecare technologies changed the daily working practices of nurses and their notions of good care [18]. Although nurses are used to seeing patients face-to-face and feared that telecare technologies might challenge their relationships with patients, they found that these technologies actually supported them in detecting patients’ problems in a timely manner.

Similar processes might occur when MS health care providers are confronted with digital self-monitoring technologies. First, because care is shifting from traditional health care settings to patients’ homes, health care providers might have to delegate some of their tasks and responsibilities to patients, such as recording measurements and interpreting data [5]. In addition, the health care provider becomes a coach who helps patients achieve their disease management goals via self-monitoring tools [14]. Second, self-monitoring can help patients gain, in principle, a better understanding of their condition. These insights can be shared with health care providers and could support their work. Although physician-patient communication could also benefit from the use of the technologies, disagreements could arise concerning whether the expertise of patients or the expertise of health care providers is the most valued [14]. Third, health care providers will have to learn how to deal with and respond to the continuous flow of data produced by digital self-monitoring [19]. The interpretation of these data can be difficult for health care providers, as they have to cope with variability in the data and decide when and how to take action in response to the data [20].

### Objectives

At the time of writing, digital self-monitoring is not common practice in MS health care. However, as several companies and public institutions, such as universities, are developing and testing smartphone apps for self-monitoring MS [7], it is appropriate to explore how health care providers envisage using these technologies in their daily work practices. To this end, we conducted interviews with neurologists, MS specialist nurses, and rehabilitation specialists in the Netherlands. These 3 groups of health care providers are the most important in the MS care ecosystem. Neurologists mainly focus on the medical aspects of the disease, such as monitoring the progress of the disease and the effectiveness of medication. MS specialist nurses have a broader perspective; they also deal with psychosocial matters, such as how the disease affects patients’ everyday lives, and are the connection between the various disciplines within the MS health care system. Most patients have appointments with their neurologist and MS specialist nurse approximately once or twice a year. Rehabilitation specialists, such as rehabilitation physicians and occupational therapists, are only visited when patients are finding it difficult to do their usual daily activities. The results of our investigations into the expectations of these health care providers can be considered in the further development and implementation of digital self-monitoring in health care.

## Methods

### Data Collection

We interviewed 14 MS health care providers: 4 neurologists, 7 MS specialist nurses, and 3 rehabilitation specialists (2 rehabilitation physicians and 1 occupational therapist) working at 3 different hospitals in the Netherlands, 1 academic hospital and 2 peripheral hospitals (Table 1). Of the 14 health care providers, 2 do not work at a hospital; they work for a care organization that was collaborating with one of the peripheral hospitals at the time of our study. The interviewed health care providers are all MS specialists, meaning that next to their general medical education they have been trained for treating patients with MS specifically.

**Table 1:** Overview of interviewed MS^a^ health care providers, including their function and the hospital (or associated care organization) they work for.

Health care provider	Function	Hospital
MS1n^b^	Neurologist	Hospital 1 (academic hospital)
MS2n	Neurologist	Hospital 1 (academic hospital)
MS3sn^c^	MS specialist nurse	Hospital 1 (academic hospital)
MS4sn	MS specialist nurse	Hospital 1 (academic hospital)
MS5r^d^	Rehabilitation physician	Hospital 1 (academic hospital)
MS6n	Neurologist	Hospital 2 (peripheral hospital)
MS7sn	MS specialist nurse	Hospital 2 (peripheral hospital)
MS8sn	MS specialist nurse	Hospital 2 (peripheral hospital)
MS9sn	MS specialist nurse	Care organization collaborating with hospital 2
MS10r	Occupational therapist	Hospital 2 (peripheral hospital)
MS11r	Rehabilitation physician	Care organization collaborating with hospital 2
MS12n	Neurologist	Hospital 3 (peripheral hospital)
MS13sn	MS specialist nurse	Hospital 3 (peripheral hospital)
MS14sn	MS specialist nurse	Hospital 3 (peripheral hospital)

^a^MS: multiple sclerosis.

^b^MSn: neurologist.

^c^MSsn: specialist nurse.

^d^MSr: rehabilitation specialist (rehabilitation physician or occupational therapist).

The interviewed health care providers were purposefully sampled and are affiliated with hospitals that at the time of the interview were planning to participate in a pilot project to test the efficiency and effectiveness of a specific smartphone app for digitally self-monitoring MS, namely, the MS Sherpa app [21]. This app contains several weekly tests, such as a walking test and a cognition test, and daily questions, about, for example, mood, energy, and stress; it aims to enable patients with MS and their health care providers to monitor patients’ mental and physical well-being. Before the interviews, we did not have any form of formal or informal collaboration with the interview respondents.

The health care providers were approached by mail by the first author (KW). For each hospital, we approached a neurologist, who subsequently connected us to the other MS health care providers working at their respective hospital. Of the 19 approached health care providers, 5 did not respond or did not want to participate in the interview. As data saturation occurred after the 14 health care providers were interviewed, meaning that no new topics or perspectives emerged, we decided not to approach additional health care providers.

The interviews were conducted between March and June 2019 and were performed by the first author KW, a PhD researcher in science and technology studies who has prior experience in conducting interviews with patients with MS and health care providers. The health care providers, who have busy schedules, could make an appointment for an interview either in person or by telephone, so as to enable some flexibility. In the end, a total of 14 interviews were conducted, 7 in person and 7 by telephone. The interviews lasted from 34 to 72 minutes, with an average duration of 56 (SD 10.08) minutes. The interview respondents received a summary of the main interview findings with the invitation to provide feedback on any false statements or other remarks, but no comments were returned.

A semistructured interview protocol was developed by the first author (KW) and the second author (LK), who is an associate professor in science and technology studies and experienced in conducting interviews with patients and health care providers about the possible effects of new biomedical innovations for their daily (work) life. The interview guide was inspired by the theme of the changing roles and responsibilities caused by digital self-monitoring, as mentioned in the *Introduction* section. The questions aim to gain an understanding of how health care providers envision digital self-monitoring of MS through smartphone apps to affect their daily working practices. Examples of interview topics were physician-patient communication, acting on self-monitoring data as a health care provider and delegating tasks and responsibilities to patients. The same interview guide was used for all interviews, but the semistructured setup of our interview guide allowed for some flexibility, for instance, to ask probing, open-ended questions on topics mentioned in previous interviews.

### Ethics Approval

The interviews were audiotaped after verbal informed consent of the respondents, which has been approved by the Research Ethics Committee of the Faculty of Science (REC19012).

### Data Analysis

First, the interviews were transcribed verbatim by the first author (KW) in Microsoft Word (2016; Microsoft Corporation). Next, both authors familiarized themselves with the data by reading the transcripts a couple of times. The transcripts were then uploaded to the qualitative data analysis software ATLAS.ti 8 (2016; ATLAS.ti Scientific Software Development GmbH) and subjected to a combination of deductive and inductive thematic content analysis [22], meaning that we aimed for systematically structuring the interview data according to the main themes and subthemes. An initial codebook was developed by the first author (KW) based on discussions with the second author (LK) and was structured according to the topics in the interview guide. The codebook was refined through iterative reading of the interview transcripts, which resulted in additional topics being added to the codebook. Final agreement on the codebook was achieved through discussion between both authors. After coding the interview transcripts, the first author (KW) started clustering the codes into subthemes, which were subsequently clustered into main themes (Textbox 1). Consensus on themes and subthemes was reached through discussion with the second author (LK). Quotes translated from Dutch (by the first author) are used throughout the *Results* section to illustrate our findings.

**Overview of the main themes and subthemes that emerged from the interviews with the health care providers.**

**Perceived value of digital self-monitoring**

- Providing additional and more complete information
- Quantifying patients’ health status

**Envisioned use of digital self-monitoring in daily working practices**

- Digital self-monitoring on prescription
- Health care providers’ access to digital self-monitoring data
- Preparation for the medical appointment
- Workload

**Delegation of tasks and responsibilities to the patient**

- More active role for patients in disease management
- Psychological and emotional burden of digital self-monitoring

## Results

### Perceived Value of Digital Self-monitoring

The interviewed health care providers expressed an ambivalent attitude toward digital self-monitoring, not only acknowledging its potential value but also expressing doubts and concerns. The longitudinal, real-world data generated by the use of smartphone apps might provide MS health care providers with information about the clinical monitoring of symptoms, disease progression, response to treatment, and side effects of medication, thereby supplementing the information collected during the medical appointment [7,8]. The interviewed health care providers indeed perceived the value of the information gained from digital self-monitoring, as they felt that information was often missing about the patient because appointments are short and there is insufficient time to perform all physical assessments, such as walking tests. Moreover, patients only have appointments once or twice a year, so it can be difficult for them to recall how their health has been over the preceding months. The health care providers believed that digital self-monitoring would produce additional and more complete information about patients’ health status. Interestingly, the health care providers perceived that the quantitative data that would be produced by digital self-monitoring would be more objective than the narratives that patients tell during an appointment:

It would provide a more objective representation of something instead of what a patient is indicating subjectively.

MS10r

There seemed to be a desire to quantify a patient’s health status to make a better assessment to complement the sensory and perceptual experiences of patients:

What I could do, is provide a better quantification. Which is often very difficult to find out during a conversation. How much is it exactly? How far can you walk? How many steps can you take? That kind of stuff. So, the quantification of all these things that we discuss should be the added value of a digital system.

MS5r

However, 2 health care providers emphasized that when there is too much of a focus on quantitative data, one runs the risk of ignoring the broader disease context, such as the psychosocial impact of MS:

If we are only occupied with measures and numbers. Will we not miss what the disease means to the patient? Not only how many meters they can walk. Because that it not always what they find the worst. But also talking about the psychosocial effect. I fear that the app misses that. That part should not be forgotten.

MS1n

### Envisioned Use of Digital Self-monitoring in Daily Working Practices

#### Digital Self-monitoring on Prescription

There are various ways in which patients can gain access to a smartphone app to self-monitor their health: they can download an app that they have found themselves and that is freely available on the commercial market, or they can use an app that has been recommended by health care providers and prescribed to patients as part of their treatment. The interviewed health care providers seemed to prefer digital self-monitoring on prescription, so that they can control which app patients use. They explained that if patients find an app themselves on the commercial market and therefore different patients use different apps, it will be difficult for health care providers to interpret the data and assess the reliability of each app. In contrast, when hospitals offer the same app that has been clinically validated to all patients, health care providers can become, over time, knowledgeable about a specific app. For instance, they can make comparisons between different patients:

I prefer to offer everyone the same app. Because if everyone uses something different, well, then it is just difficult to keep track of it. How reliable it is what they are doing. If something has been standardized and validated for what you are measuring. Then you can indeed offer it as a standard to patients so that you also get used to the numbers.

MS1n

This quote suggests that health care providers like to stay in charge when it comes to the digital self-monitoring of MS. They stressed that offering a smartphone app to patients should be an optional part of hospital treatment and only considered when the health care provider believes it could be valuable for the patient and when the patient is interested. As the health care providers explained, some patients are enthusiastic about collecting their personal health data, but others are not. Therefore, multiple health care providers emphasized that they have to consider whether digital self-monitoring is suitable for a specific patient. Health problems, such as cognitive or physical dysfunctions or limited (digital) health literacy, might hinder the use of digital self-monitoring by patients with MS. When health care providers are deciding which patients will be prescribed an app, they are at risk of becoming gatekeepers who control patients’ access to digital self-monitoring.

#### Health Care Providers’ Access to Digital Self-monitoring Data

Patients’ digital self-monitoring raises a question about who should be able to access the collected data: the patient, the health care provider, or both. A common belief among the interviewed health care providers was that patients should control who has access to these data. All respondents agreed that these data should be accessible to patients and that the idea that patients cannot view their own health data is old-fashioned:

Well, these are the patient’s data. So at the current time I do not think you can say that these are not available to them.

MS2n

The health care providers believed that they should only have access to the self-monitoring data after the patient had consented to this access. They envisioned that patients could select the data they wanted to be accessible to a particular health care provider. Most of the health care providers thought that it would be valuable to have access to the self-monitoring data. However, 2 health care providers expressed some doubts about patients’ data becoming available to them, as this quote illustrates:

That would be sort of a Big Brother—that I can see from my computer how all my patients are doing. I am a bit hesitant about that.

MS5r

Having easy access to the self-monitoring data was crucial to the health care providers, as they have limited time to prepare for each appointment. The health care providers thought that a significant barrier is created when access to self-monitoring data is not straightforward, for example, because it involves logging in on a different system. As a respondent explained, their hospital had stopped using a web-based portal for digital self-monitoring because it took too much effort to access the data:

We have used [name of web-based portal] for a while. But it was just not workable. It was all external. And then we have to open it next to our file and that is just really difficult. That we have to go to their site to access patients’ data. Well, if I have to do this in my half hour slot, I will not make it.

MS7sn

All health care providers preferred the digital self-monitoring data to be integrated into the patient’s electronic file, because this made accessing the data easy. However, they also acknowledged that this would be technically complicated, as systems are often not compatible with each other.

#### Preparation for the Medical Appointment

The health care providers expected that they would mainly use the digital self-monitoring data to prepare for an appointment with a patient, so that they could focus on the patient’s most prominent problems:

I think you can make your conversation more to the point. That you can leave out some domains where there are no problems. So I can use the remaining conversation time to focus on the areas where there are problems and that the patient has questions about. So I think it results in more directed conversations during the medical appointment.

MS5r

Although previous research has shown that patients feel reassured when health care providers monitor their health data in between appointments [23,24], our findings show that, because of a lack of time, the health care providers did not expect that they would look at the self-monitoring data in between appointments, unless the app or the patient indicated that something may be wrong. This suggests that health care providers do not think that it is their responsibility to monitor these data to detect deviations from the expected results or deterioration in the condition.

Moreover, the health care providers thought that patients could use smartphone apps to indicate the topics they wanted to talk about, but this would require patients to be willing and able to take the lead when engaging in digital self-monitoring. As one of the respondents explained, this would allow an appointment to be adapted to an individual patient’s needs:

Tomorrow you have a check-up with the rehabilitation physician. What would you like to discuss? If the patient submits that, I can prepare better. Now I notice that patients are saying: well, I wanted to discuss this and this, but there was no time. Such an app could help with that.

MS11r

#### Workload

The interviewed health care providers were ambivalent about the impact that digital self-monitoring might have on their workload. On the one hand, they imagined that it could result in a more efficient workflow as the data could make the appointment with the patient more efficient. On the other hand, they stated that digital self-monitoring requires a new way of working, such as integrating the data into daily workflows. They thought that this would take additional time, especially at the beginning. Moreover, some health care providers were worried that digital self-monitoring might result in information overload.

Be careful that you do not get so much information that you drown in information that is not useful. Not so much information that you lose the overview completely. You have to be careful that it does not become overkill.

MS6n

### Delegation of Tasks and Responsibilities to Patients

Digital self-monitoring requires that health care providers, to a certain extent, take on the role of *coach at a distance*, as tasks such as collecting and interpreting data are delegated to the patient. Patients are expected to become proactive in the management of their own medical care and lifestyle. However, the health care providers indicated that much responsibility is already given to the patient in the management of MS and that patients with MS tend to take on an active role:

I think that among the patient groups, MS patients already have and take quite an active role. My experience is that most patients are quite willing to take charge.

MS6n

The health care providers believed that digital self-monitoring could help patients with MS to have even more control. They imagined that it would enable patients with MS to follow the progress of their disease, find patterns in their personal data, and compare these data with their personal experiences:

What could be an aim, is that through time they can see what is happening with them: days that they are feeling better or worse; what influence this has on the things that they are tracking.

MS1n

I think it can provide insight to patients about when symptoms are occurring and what kind of symptoms. Maybe it becomes easier to make connections between certain things.

MS3sn

According to some health care providers, patients could act on these insights, for instance, by contacting the health care provider if necessary. They also mentioned how smartphone apps could support patients in their particular lifestyle, such as stimulating them to be physically active:

Well, I think that the motivation for a good lifestyle, for instance the motivation to increase movement, that it works for many people. That thing [the smartphone app] provides compliments. If you are the type for that and you are sensitive to that, then it works very well.

MS9sn

Although the health care providers acknowledged the potential value of digital self-monitoring in terms of empowering patients with MS, they also expressed concerns regarding the reconfiguration of patients’ roles and responsibilities. The health care providers referred to the potential psychological and emotional burden of digital self-monitoring. They explained that digital self-monitoring might confront patients with their disease and make them feel more like a patient. Multiple health care providers mentioned that because of their experiences with patients with MS, they know that many of them do not like to be preoccupied with their disease:

I think that also a lot of patients will say: I do not want to be confronted with my disease, so I am not going to use it. Right now I have some patients who are saying: I do not want to be confronted with my disease. I just want to live.

MS7sn

Moreover, the health care providers were concerned that digital self-monitoring data might worry or disappoint patients, for instance, because there are signs that the disease is progressing. This suggests that health care providers are ambivalent about the reconfiguration of patients’ roles and responsibilities.

## Discussion

### Principal Findings

At the time of writing, several smartphone apps that digitally self-monitor MS are being developed [7], which creates a novel situation in which health care providers and patients must engage in a reconfiguration of roles and responsibilities. From our interviews, we have gained insight into the expected new configuration from the perspective of MS health care providers. The health care providers in our study are generally willing to use smartphone apps for self-monitoring of patients and value the quantified data that become available because they complement patients’ narratives that are revealed during medical appointments. We found that the health care providers primarily want to use digital self-monitoring on prescription, meaning that they want a standardized smartphone app that has been clinically validated. They also believed that digital self-monitoring should always be an option, rather than an obligation. They acknowledged that not all patients are willing or able to engage in digital self-monitoring and expressed concerns regarding the potential psychological and emotional burden of this technology. Furthermore, the health care providers envisioned a delegation of particular tasks and responsibilities to patients, such as sharing data with their health care provider and contacting the hospital if necessary. However, this new configuration would potentially bring with it considerable tensions and issues regarding the type of patient empowerment and personalized health care that the digital self-monitoring is aiming for, and we describe the main four next.

First, the prescription of self-monitoring apps by health care providers is in line with the idea of *clinical self-tracking* in which self-monitoring practices are pushed by health care providers, that is, self-monitoring by the patient for therapeutic purposes following the recommendation of health care providers [25]. In these clinical self-tracking practices, data are shared with health care providers, and data collection is standardized to help health care providers analyze and interpret the data. However, if health care providers act as gatekeepers regarding patients’ access to digital self-monitoring and standardized apps are used, this could restrict patients’ autonomy and reinforce the *paternalistic model* in which health care providers rather than patients are in charge [26]. Will there be time during medical appointments for patients to discuss data produced by self-monitoring apps that they have purchased themselves on the commercial market, for example? Moreover, our previous research showed that patients with MS want their use of digital self-monitoring to be flexible, that is, to consider their personal situation and disease status [12]. However, because of what health care providers said in the interviews, we wonder whether patients will have the flexibility to deviate from self-monitoring protocols and whether, if they do so, health care providers might then distrust the data. Therefore, when self-monitoring apps are prescribed by health care providers, there might be a tension between the traditional paternalistic notion of physician-patient relationships and the ideal of patient empowerment, that is, patients being in charge of their own disease management, as promoted by the developers of these apps. When opting for a specific implementation strategy, such as self-monitoring on prescription, technology developers should consider that this implies a certain vision on health care.

Second, how realistic is it to think that patients will engage in the tasks and responsibilities as imagined by the health care providers in our study? Discrepancies can exist between the expectations of health care providers and the assumptions of patients regarding their roles and responsibilities. Although the health care providers in our study thought that patients would contact them if there were deviations in the self-monitoring data, previous research has found that patients are hesitant to do so as they downgrade their problems and do not want to bother their health care providers [27]. Furthermore, tensions can arise when patients and health care providers have different expectations about the amount of support that health care providers offer in the self-monitoring process [28]. Patients might feel reassured by a sense of being supervised by their health care providers [23,24], but the health care providers in our study stressed the personal responsibility of patients in the management of their disease, for instance, their responsibility for signaling potential signs of disease progression. The promises and expectations surrounding digital self-monitoring, as among others expressed by technology developers, are based on ideal situations and assume that patients have the right knowledge and skills to deal with the collected data and are willing and able to engage in self-managing their health [1,14]. However, as noted previously, patients’ physical and cognitive capacities, and limited digital or health literacy, could pose a barrier to patients’ abilities to adapt to the new roles and responsibilities required by digital self-monitoring, such as correctly interpreting their own data and reacting appropriately to it [29]. Moreover, digital self-monitoring could pose a psychological and emotional burden on patients, which might have a disempowering rather than an empowering effect on patients [1,2,30]. These factors imply that not all patients are willing or able to engage in digital self-monitoring, which might result in health inequities when self-monitoring apps become an integral part of health care [29]. In fact, as Prainsack [31] already argued, the introduction of technologies to personalize health care, such as self-monitoring apps, as well as genetic self-tests and home-based blood monitoring technologies, might confront us with fundamental questions such as what social circumstances and capacities do patients need to meet to participate in the shift toward more personalized, digital health care? In addition, which groups of patients tend to be included and excluded when digital self-monitoring gains more significance in health care?

Third, when digital self-monitoring reduces health to data and algorithms, the richness and complexity of patients’ experiences can be lost [2]. Tensions might arise between the place that objective numbers will be given in health care and patients’ subjective narratives [31]. The interviewed health care providers seemed to want quantitative assessments of patients’ health status to complement patients’ experiences. However, as 2 health care providers also acknowledged, this might result in too much of a focus on numbers while the broader context of the patient, such as their psychological struggles, is ignored [2]. When the conversation is dominated by the quantitative data during the medical appointment, patients have less time to share their personal stories. Consequently, the questions and issues that are the most pressing ones for the patient might not be discussed. Moreover, it is questionable how objective these quantitative data truly are. Although numbers seem objective and neutral, they are full of assumptions and value judgments, such as choices made regarding which parameters are being measured and how [1,2]. There might also be discrepancies between what the numbers are indicating and what the patients are experiencing. This brings us to the sociological concepts of *illness* and *disease*, with illness being defined as how patients identify with their ill-health and disease meaning the condition diagnosed by a health care provider, that is, as biologically defined [32]. When health care providers value quantitative self-monitoring data more than other factors, they may primarily focus on the biological condition, that is, the disease, whereas we know that illness is just as important for patients. These issues highlight that when integrating digital self-monitoring data into health care, one needs to consider how to balance quantitative data with patients’ feelings and experiences when judging a patient’s health status. This discussion is interesting in the light of the current paradigm shift toward *personalized health care* [2]. Our findings bring the following question to the fore: what makes personalized health care really personalized? Does personalized health care involve having more quantitative data on patients’ health status, or is it about paying more attention to patients’ personal experiences? Alternatively, should both elements be combined to achieve truly personalized health care? We recommend that the developers of digital self-monitoring technologies be aware of the fact that personalized health care is not a straightforward concept and can evoke different connotations for patients, health care providers, and technology developers.

Finally, although the health care providers in our study valued digital self-monitoring of MS, our findings also underline that is not self-evident that health care providers are willing and able to use this technology as part of their daily practices. The new roles and responsibilities demanded by the use of digital self-monitoring, such as delegating the collection, interpretation, and sharing of data to patients, can be a challenge for health care providers and their professional identities and routines as our study shows. Moreover, as the literature on mobile health technologies for other disease domains has already highlighted, health care providers also experience other barriers, which are related to the quality, validity, accuracy, and clinical utility of mobile health technologies and patient-generated data [33-35]. As several studies have shown, health care providers have more confidence in data collected by themselves than by patients [30,34,36]. Furthermore, health care providers have raised medical legal concerns about data security, privacy, and accountability, such as the confidentiality of self-monitoring data and the responsibility of health care providers to act upon patient data [34,37].

These considerations illustrate the complexities surrounding the use of digital self-monitoring technologies by health care providers and also indicate that the embedding of these technologies in clinical practices cannot be decided by using a top-down approach. Instead, a mutual collaboration is needed between the technology developers and the health care providers who are expected to work with digital self-monitoring. There often seems to be a mismatch between the developers of mobile health technologies and the health care providers who ought to use these technologies, with insufficient consideration being given to user needs [37,38]. As Tarricone et al [37] argue, app developers should do more to include health care providers during the development process of health apps. When the developers of digital self-monitoring technologies adopt such an approach, implementation strategies for digital self-monitoring can be developed iteratively so that there is a greater chance that the technology fits the daily working practices of health care providers.

### Study Limitations

As far as we know, this is the first qualitative study on the expectations, desires, and concerns of MS health care providers regarding digital self-monitoring. Although this study has provided a rich insight into the perspectives of health care providers, future studies would probably benefit from including more respondents per health care provider group (neurologists, specialist nurses, and rehabilitation specialists). This would allow for a more rigorous comparison between these groups, which goes beyond the aim of this study. In addition, other health care providers involved in the care of patients with MS, such as psychologists and physiotherapists, could be included in future studies.

Moreover, although we focused on MS specialists, as these respondents were thought to be the most informative for this study, not all patients with MS are treated by health care providers specializing in MS. In fact, many patients with MS are under treatment of general neurologists and rehabilitation specialists. It would be interesting to study the similarities and differences in needs, wishes, and experiences regarding digital self-monitoring of MS between the MS specialists and the general health care providers.

Another consideration we would like to point out is that the interviewed health care providers are affiliated with hospitals that will be enrolled in a pilot study on digital self-monitoring of MS. These health care providers might therefore have a biased positive attitude toward the value of digital self-monitoring compared with those who are not going to be engaged in the study. Finally, our study only investigated Dutch health care providers’ attitudes. It might be interesting to compare our findings with the attitudes of health care providers in other countries because of cultural differences in different countries’ health care systems.

### Conclusions

To conclude, in this study, we have identified potential tensions and problems that might occur when digital self-monitoring is introduced in existing health care. Our findings could be considered in study protocols of future projects on digital self-monitoring or in implementation strategies for integrating these technologies into clinical practices. For instance, developers could consider what type of personalized health care and patient empowerment they want to stimulate with their technology, as this is not straightforward and patients and health care providers can have different visions in this regard. As there might be differences between what people say and the actual acts they perform, in future research, it would be very interesting to investigate the actual actions and experiences of health care providers with regard to digital self-monitoring to determine how the tensions mentioned in this paper are being played out in practice.

## Abbreviations

MS: multiple sclerosis
